# Effects of Galvanic Vestibular Stimulation on Visual Verticality and Standing Posture Differ Based on the Polarity of the Stimulation and Hemispheric Lesion Side in Patients With Stroke

**DOI:** 10.3389/fneur.2021.768663

**Published:** 2021-11-11

**Authors:** Takamichi Tohyama, Kunitsugu Kondo, Yohei Otaka

**Affiliations:** ^1^Department of Rehabilitation Medicine, Tokyo Bay Rehabilitation Hospital, Narashino, Japan; ^2^Department of Rehabilitation Medicine I, School of Medicine, Fujita Health University, Toyoake, Japan

**Keywords:** cerebrovascular disorder, hemiparesis, postural balance, subjective visual vertical, vestibular control

## Abstract

**Introduction:** There is growing evidence supporting the relationship of vertical misperception and poor balance control with asymmetrical standing posture in patients with stroke. Although the vestibular system has been shown to be responsible for vertical misperception and balance disorders, the effect of galvanic vestibular stimulation (GVS) on both vertical misperception and postural asymmetry after stroke remains elusive. The aim of this study was to investigate the effects of GVS on visual verticality and postural asymmetry after stroke and to clarify whether the effects differ depending on the polarity of the stimulation and hemispheric lesion side.

**Methods:** We measured the subjective visual vertical (SVV) and body weight distribution on each foot in an upright stance in 24 patients with a hemispheric stroke (10 with a left hemisphere lesion and 14 with a right hemisphere lesion) and nine age-matched healthy controls. During the measurements, bipolar GVS (1.5 mA) was applied over the bilateral mastoid processes in three stimulation conditions: contralesional-anodal and ipsilesional-cathodal vestibular stimulation, ipsilesional-anodal and contralesional-cathodal vestibular stimulation, and no stimulation. To examine whether GVS modulates visual verticality and standing posture, SVV and weight-bearing in the three conditions were analyzed.

**Results:** During no stimulation, the SVV deviated to the contralesional side in patients with a right hemisphere lesion, while more weight-bearing was observed on the ipsilesional limb than on the contralesional limb in both patient groups than in the controls. The SVV was modulated by reversing the polarity of GVS in all the groups when the cathodal stimulus side was either ipsilateral or contralateral to the lesion while the ipsilesional-cathodal vestibular stimulation reduced weight-bearing asymmetry in only the patients with a right hemisphere lesion.

**Conclusions:** These findings demonstrate that the effects of GVS on the SVV and standing posture differ depending on the polarity of GVS and the hemispheric lesion side. Patients with a right hemisphere lesion have difficulty maintaining their preferred standing posture under visual verticality modulation evoked by GVS. The application of GVS may clarify whether the vestibular system has neural redundancy after stroke to suppress any effects of the stimulation, including modulation of the visual verticality, on balance.

## Introduction

Postural control to maintain upright posture is impaired in stroke survivors (1). The standing posture of patients with hemiparetic stroke is characterized by more weight-bearing on the ipsilesional limb than on the contralesional limb and by postural instability (2, 3). There is growing evidence that deviation of the subjective vertical is related to asymmetrical standing posture (4, 5), poor balance control (6), and poor recovery of balance in patients with stroke (7). Particularly, in patients with a right hemisphere lesion, poor recovery of the deviation of the subjective visual vertical (SVV), poor postural control, and the presence of visuospatial neglect were closely related to each other (8–11). Based on lesional and imaging studies in patients with peripheral vestibular lesions or stroke, it has been proposed that a dysfunction of the vestibular system can cause misperception of the SVV and postural disorders (12). However, whether manipulation of the vestibular system can change the misperception of the visual vertical and asymmetrical postural control after stroke is unclear.

Galvanic vestibular stimulation (GVS) can increase or decrease the firing rate of vestibular afferents by reversing the polarity; the cathodal galvanic stimulation results in excitation, and the anodal galvanic stimulation results in inhibition of the vestibular afferents through the spike trigger zone of primary afferents (13). GVS has been an easily applicable tool in clinical and therapeutic investigations over the last decade (14). However, only a few studies (15, 16) have investigated the effects of GVS on verticality after stroke, reporting that contralesional-cathodal vestibular stimulation reduced the pathological deviation of the SVV in patients with a right hemisphere stroke who have visuospatial neglect. Although one study observed that cold caloric vestibular stimulation reduced postural asymmetry when patients stood spontaneously (17), no study has clarified the effects of GVS on their preferred weight-bearing asymmetry. Bilateral bipolar GVS, delivered with a cathodal electrode on the mastoid process behind one ear and an anodal electrode on the other ear, produces mediolateral postural sway in healthy subjects (18), which can appear as the sum of the vestibular organs responses (19). Therefore, the potential effects of bipolar GVS on both visual verticality and asymmetrical standing posture in patients with stroke remain elusive. In addition, it is unclear if these potential effects differ depending on the polarity of the stimulation and hemispheric lesion side.

The aim of this study was to investigate the effects of bipolar GVS on visual vertical perception and asymmetrical standing posture after stroke and to clarify whether the effects differ depending on the polarity of the stimulation and hemispheric lesion side.

## Materials and Methods

### Participants

Patients admitted to Tokyo Bay Rehabilitation Hospital, Narashino, Japan, were included in this study if they developed a new single cerebral stroke lesion, understood verbal instructions, and were able to stand independently without the use of any orthosis or aids at the time of the study. Patients were excluded if they had a history of stroke, other neurological diseases, or orthopedic impairments affecting an upright stance. Twenty-four patients, of whom 14 had a right hemisphere stroke lesion and 10 had a left hemisphere stroke lesion, participated in this study. All the patients underwent a conventional rehabilitation program of physical therapy, occupational therapy, and speech therapy if needed. The brains of the patients were scanned using computed tomography or magnetic resonance imaging. The locations of lesions were classified using the Talairach and Tournoux atlas (20). Nine age-matched healthy individuals without any neurological or orthopedic impairments that could affect an upright stance were recruited as controls. The characteristics of the patients are shown in Table 1 and summarized in Table 2. The purpose and procedures of the study were explained to the participants, and they provided their written informed consent. The study protocol was reviewed and approved by the local ethics committee.

**Table 1:** Characteristics of the patients with stroke.

**Patient**	**Age/Sex**	**Handedness**	**Etiology**	**Lesion**		**Time from lesion** **(days)**	**Stroke Impairment Assessment Set**		
				**Location**	**Side**		**Motor score**	**Sensory score**	**Visuospatial score**
1	67/m	R	H	Th	R	83	10	3	3
2	72/m	R	I	F/Rc/P/C/T	R	141	0	0	2
3	55/m	R	I	F/Rc/P/C/S/Ic/T	R	157	4	0	1
4	78/f	R	I	C	R	104	7	2	3
5	64/m	R	I	C/F/S	R	97	12	3	2
6	61/m	R	I	C/F/S/T/Rc	R	144	4	2	3
7	74/m	R	H	Th	R	104	12	3	3
8	66/m	R	H	C/Th	R	148	9	2	3
9	65/m	R	I	Ic/C/F	R	99	15	3	3
10	79/m	R	I	C	R	57	10	3	2
11	47/m	R	H	C/S/Ic	R	141	8	1	3
12	70/f	R	I	F/T	R	123	15	3	3
13	70/m	R	I	C	R	76	8	3	3
14	55/m	R	I	C/S	R	40	9	3	3
15	64/m	R	I	C/S	L	113	12	3	3
16	76/m	R	I	F/P	L	163	1	2	n/a
17	54/m	R	H	C/S/Ic	L	80	3	1	3
18	39/m	R	I	F	L	48	15	3	3
19	64/f	R	I	F/T	L	46	15	2	3
20	69/m	R	I	F	L	33	15	3	3
21	64/f	R	H	C/S	L	72	11	3	3
22	64/f	R	H	C/S	L	92	11	3	3
23	42/m	L	H	F/C/S	L	56	12	3	3
24	66/m	L	H	C/S/Ic	L	65	7	3	3

*m, male; f, female; R, right; L, left; H, hemorrhagic; I, ischemic; Th, thalamus; F, frontal cortex; Rc, Rolando's cortex; P, parietal cortex; C, corona radiata; T, temporal cortex; S, striatum; Ic, internal capsule; n/a, not available*.

**Table 2:** Summary of the characteristics of the controls and patients with stroke.

**Variables**	**Controls (*n* = 9)**	**Stroke**		***P***
		**Left hemisphere lesion (*n* = 10)**	**Right hemisphere lesion (*n* = 14)**	
Age, years, mean (SD)	60.6 (5.7)	60.2 (11.7)	65.9 (9.1)	0.249^*^
Sex, male/female, *n*	4/5	7/3	12/2	0.116^†^
Handedness, right/left, *n*	8/1	8/2	14/0	0.244^†^
Etiology, hemorrhagic/ischemic, *n*	–	5/5	4/10	0.402^†^
Time from stroke onset, days, mean (SD)	–	76.8 (38.4)	108.1 (36.0)	0.052^‡^
Stroke Impairment Assessment Set				
Motor score (range: 0–15), median (IQR)	–	11.5 (8)	9 (5)	0.345^a^
Sensory score (range: 0–3), median (IQR)	–	3 (1)	3 (1)	0.456^§^
Visuospatial score (range: 0–3), median (IQR)	–	3 (0)	3 (0)	0.273^§^

*SD, standard deviation; IQR, interquartile range*.

* *Comparisons using a one-way analysis of variance (ANOVA)*.

† *Comparisons using the Fisher's exact test*.

‡ *A comparison using the two-sample t-test*.

§ *A comparison using the Wilcoxon rank sum test*.

### Clinical Assessment

We performed neurological examinations before the SVV and standing posture assessment. The following impairments were evaluated: hemiparesis using the summed motor item scores of the Stroke Impairment Assessment Set (SIAS) for the hip, knee, and ankle joint (range of each joint score: 0–5, where 0 indicates no muscle contraction, and 5 indicates limb movement that is as fast as the non-paretic limb) (21, 22), proprioception of the great toe using the position sensation score of the lower limb sensory item of the SIAS (range: 0–3, where 0 indicates no sense of movement on the great toe, and 3 indicates small changes in the position of the great toe that can be perceived as exactly like that of the non-paretic limb), and visuospatial perception using the visuospatial score of the SIAS (range: 0–3, where 0 indicates more than 15 cm deviation from the mid-point when bisecting a 50-cm line, and 3 indicates <2 cm deviation from the mid-point).

### Assessment of SVV Perception

The SVV was measured with participants seated on a chair in front of a monitor covered with a cylinder (diameter, 25 cm; height, 25 cm) to eliminate any visual reference cues. The participants could look at the monitor through the cylinder using binoculars. A white line (7 × 0.5 cm) was projected onto the black background of the monitor in a pseudo-random oblique position. An examiner rotated the line around its center until the participants perceived it as vertical. The rotation angle formed by the subjective vertical and gravitational vertical lines was measured. The direction to the ipsilesional side (for the controls, the right side, i.e., clockwise rotation, was used) was set as positive. The mean SVV value of eight trials in each session was calculated and used for further analysis.

### Assessment of Standing Posture

To evaluate the standing posture of the participants, bodyweight distribution was measured by two rectangular force platforms (G-7100; Anima; Tokyo, Japan) placed side by side. The participants stood barefoot with each foot placed on one of the two platforms (heels separated by 9 cm, toe out at 30°), arms relaxed and hanging freely along the body, and eyes opened, looking straight ahead at a fixed target. The participants were instructed to stand for 30 s during a recording of a trial. For three trial recordings, with short resting intervals between the trials, no feedback or information was given to the participants. Postural asymmetry was evaluated using the weight-bearing ratio (WBR), expressed as the percentage of the total body weight loading the ipsilesional side (for the controls, the right side was used). The mean WBR of the three trials in each session was used for further analysis. More than 50% of WBR indicates more weight-bearing on the ipsilesional limb than on the contralesional limb (for the controls, more than 50% on the right side than on the left side).

### Galvanic Vestibular Stimulation

Direct current was delivered to a pair of self-adhesive electrodes (35 mm in diameter) made of Ag/AgCl placed over the bilateral mastoid processes using an electrical stimulator (SEN-3301; Nihon Kohden; Tokyo, Japan). The participants received bipolar GVS in three conditions: contralesional-anodal and ipsilesional-cathodal vestibular stimulation (ipsiVS), ipsilesional-anodal and contralesional-cathodal vestibular stimulation (contraVS), and no stimulation. The abbreviations were determined based on the side of the excitation. The participants first performed the tasks for the SVV and WBR measurements, with electrodes in place without the stimulation. They then performed the tasks again with bipolar GVS. They were blinded to the type of stimulation being delivered. The intensity of the current was gradually increased from zero to 1.5 mA. During the tasks, all participants received 1.5 mA constant bipolar GVS. During the SVV task, a vestibular stimulation was applied in eight trials in a session. During the standing posture task, a vestibular stimulation for 30 s was applied in a trial. The standing posture trial was repeated a total of three times in a session. The SVV task and the standing posture task on the same stimulation were performed in the same day. The time interval between the ipsiVS and the contraVS was 1–3 days. The controls received vestibular stimulation with the same procedures as that of the patients with stroke [left-anodal and right-cathodal vestibular stimulation (rtVS), right-anodal and left-cathodal vestibular stimulation (ltVS), and no stimulation]. For the readability of the names of stimulation conditions, only the cathodal stimulus side has been presented from here.

### Statistical Analysis

The normality of the data was examined using the Kolmogorov-Smirnov test. The Bartlett test was performed to examine the equality of variances between the groups. Comparisons of the clinical backgrounds of patients with a left hemisphere lesion and those with a right hemisphere lesion were performed using the Fisher's exact test, the two-sample *t*-test, or the Wilcoxon rank sum test according to the type of variable. The comparisons of the SVV or WBR during no stimulation between the controls and the patients with stroke were performed using a one-way analysis of variance (ANOVA) or the Kruskal-Wallis test according to the normality and equality of variances of the data. The comparisons of the SVV or WBR among the types of bipolar GVS within each group were performed using a one-way ANOVA with repeated measures. The Greenhouse-Geisser correction was used if sphericity was not met. *Post-hoc* pairwise comparison tests were performed using Bonferroni's method. To measure the absolute effect size of bipolar GVS within each group, Cohen's *d*-value between the two stimulation conditions (ipsiVS and contraVS for the patients with stroke; rtVS and ltVS for the controls) was calculated. Classification of the effect size was based on the Cohen's criteria (23). All tests were performed using MATLAB R2020a (Mathworks, Natick, MA, USA) or R version 3.5.3 (2019-03-11) (The R Foundation for Statistical Computing, Vienna, Austria. URL https://www.R-project.org/). A *p* < 0.05 was considered statistically significant.

## Results

### Characteristics of the Controls and the Patients With Stroke

The demographics and clinical details of the patients are shown in Table 1 and summarized in Table 2. There were no significant differences in age, sex, and handedness between the groups (all *p* > 0.05). Mean (SD) time from stroke onset in the patients with a right hemisphere lesion was 108.1 (36.0) days and tended to be longer than that in the patients with a left hemisphere lesion (mean [SD], 76.8 [38.4] days; *p* = 0.052). No significant differences in the severity of the motor impairments (*p* = 0.345) and sensory function (*p* = 0.456) of the lower limbs and visuospatial function (*p* = 0.273) were found between patients with a left hemisphere lesion and those with a right hemisphere lesion.

### Visual Verticality and Weight-Bearing Without Stimulation in Three Groups

Between-group comparisons of the SVV and WBR during no stimulation are shown in Table 3. The SVV was more significantly deviated (*p* = 0.048) to the contralesional side in the patients with a right hemisphere lesion (mean [SD], −1.0° [2.5°]) than in the controls (0.7° [1.0°]), suggesting that the patients with a right hemisphere lesion misperceived the gravitational vertical toward the contralesional side. There was no significant difference in the SVV between the patients with a left hemisphere lesion (−0.5° [1.5°]) and the controls (*p* = 0.208). Contrarily, asymmetry in weight-bearing was found in the patients with a right hemisphere (mean [SD], 59.6% [12.2%]) and a left hemisphere lesion (58.0% [8.7%]) compared with that found in the controls (46.1% [5.6%]) (*p* = 0.009 and *p* = 0.038, respectively); weight-bearing was more on the ipsilesional (non-paretic) limb than on the contralesional limb. There was no difference in weight-bearing between the patients with a right hemisphere lesion and those with a left hemisphere lesion (*p* = 0.999).

**Table 3:** Visual verticality and weight-bearing without stimulation in three groups.

**Variables**	**Controls (*n* = 9)**	**Stroke**		***P***	***P***
		**Left hemisphere lesion**	**Right hemisphere lesion**	**ANOVA**	***Post-hoc***
		**(*n* = 10)**	**(*n* = 14)**		
Subjective visual vertical, degree, mean (SD)	0.7 (1.0)	−0.5 (1.5)	−1.0 (2.5)	0.047	C vs. L: 0.208 C vs. R: 0.048 L vs. R: 0.999
Weight-bearing ratio, %, mean (SD)	46.1 (5.6)	58.0 (8.7)	59.6 (12.2)	0.007	C vs. L: 0.038 C vs. R: 0.009 L vs. R: 0.999

*SD, standard deviation; C, controls; L, left hemisphere lesion; R, right hemisphere lesion*.

### Effects of Bipolar Galvanic Vestibular Stimulation on the Visual Verticality in Three Groups

**Figure 1:**
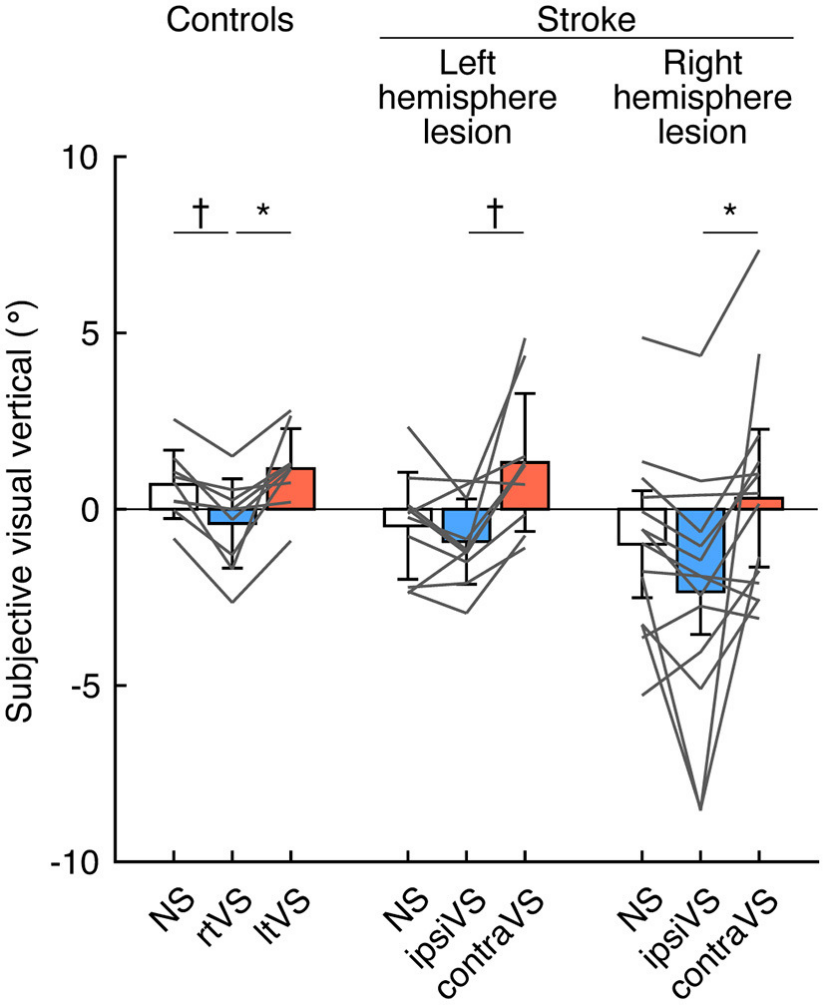
Effects of bipolar galvanic vestibular stimulation on the visual verticality in the controls, patients with a left hemisphere lesion, and patients with a right hemisphere lesion. Each color bar indicates the mean of the subjective visual vertical (SVV) during each type of bipolar galvanic vestibular stimulation (GVS). Positive values indicate rotations to the right side (for the controls) and the ipsilesional side (for the patients with stroke). Each error bar indicates a standard deviation. Each gray line indicates changes in the SVV during each type of bipolar GVS in each participant. NS, no stimulation; rtVS, left-anodal and right-cathodal vestibular stimulation; ltVS, right-anodal and left-cathodal vestibular stimulation; ipsiVS, contralesional-anodal and ipsilesional-cathodal vestibular stimulation; contraVS, ipsilesional-anodal and contralesional-cathodal vestibular stimulation. *p < 0.05; ^*†*^*p* < 0.01.

To examine the within-group differences of bipolar GVS effects on visual verticality, the SVV was compared among the types of stimulation in each group (Figure 1). There were significant differences in the SVV among the types of stimulation in all the groups: controls [*F*_(2, 16)_ = 12.49, *p* < 0.001], patients with a left hemisphere lesion [*F*_(2, 18)_ = 10.48, *p* < 0.001], and patients with a right hemisphere lesion [*F*_(2, 26)_ = 6.92, *p* = 0.014]. *Post-hoc* analyses revealed that the SVV during the rtVS was more significantly deviated to the left side compared to the SVV during no stimulation and the ltVS in the controls (*p* = 0.008 and *p* = 0.018, respectively) (Figure 1; left). Similarly, the SVV more significantly deviated to the contralesional side during the ipsiVS than during the contraVS in patients with both left hemisphere lesion or right hemisphere lesion (*p* = 0.008 and *p* = 0.048, respectively) (Figure 1; middle and right). The effect size was large in the controls (Cohen's *d* = 1.23) and in the patients with a left hemisphere lesion (Cohen's *d* = 1.28). In addition, the effect size was almost large in the patients with a right hemisphere lesion (Cohen's *d* = 0.74).

### Effects of Bipolar Galvanic Vestibular Stimulation on Weight-Bearing in Three Groups

**Figure 2:**
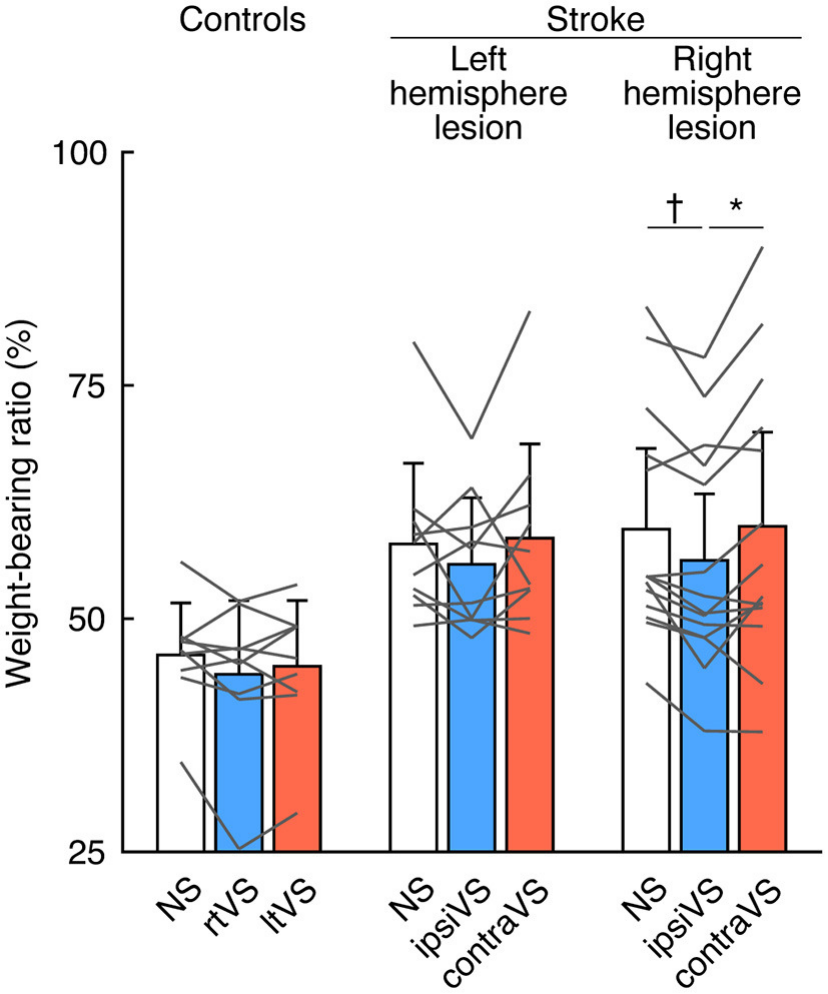
Effects of bipolar galvanic vestibular stimulation on weight-bearing in the controls, patients with a left hemisphere lesion, and patients with a right hemisphere lesion. Each color bar indicates the mean weight-bearing ratio (WBR) during each type of bipolar galvanic vestibular stimulation (GVS). More than 50% of WBR indicates more weight-bearing on the right side (for the controls) and the ipsilesional side (for the patients with stroke). Each error bar indicates a standard deviation. Each gray line indicates changes in the WBR during each type of bipolar GVS in each participant. NS, no stimulation; rtVS, left-anodal and right-cathodal vestibular stimulation; ltVS, right-anodal and left-cathodal vestibular stimulation; ipsiVS, contralesional-anodal and ipsilesional-cathodal vestibular stimulation; contraVS, ipsilesional-anodal and contralesional-cathodal vestibular stimulation. *p < 0.05; ^*†*^*p* < 0.01.

To examine the within-group differences of bipolar GVS effects on standing posture, the WBR was compared among the types of stimulation in each group (Figure 2). There were no differences in weight-bearing among the types of stimulation in the controls [*F*_(2, 16)_ = 1.96, *p* = 0.173] and the patients with a left hemisphere lesion [*F*_(2, 18)_ = 1.55, *p* = 0.245] (Figure 2; left and middle). The effect size was small in the controls (Cohen's *d* = 0.32) and in the patients with a left hemisphere lesion (Cohen's *d* = 0.41). However, there was a significant difference in weight-bearing in the patients with a right hemisphere lesion [*F*_(2, 26)_ = 6.36, *p* = 0.005]; the ipsiVS significantly reduced the asymmetry in weight-bearing compared with that by no stimulation and the contraVS (*p* = 0.007 and *p* = 0.039, respectively) (Figure 2; right). The effect size was almost large in the patients with a right hemisphere lesion (Cohen's *d* = 0.77).

## Discussion

We examined the effects of bipolar GVS on both visual verticality and standing posture in patients with hemispheric stroke, and whether the effects differ depending on the polarity of the stimulation and hemispheric lesion side. We obtained the following results: (i) modulation of the visual verticality by reversing the polarity of the stimulation in all the groups when the cathodal stimulus side was either ipsilateral or contralateral to the lesion and (ii) a shift of weight-bearing from the ipsilesional limb to the contralesional limb; reduction of weight-bearing asymmetry during the ipsilesional-cathodal vestibular stimulation compared to no stimulation and the contralesional-cathodal vestibular stimulation in the patients with a right hemisphere stroke only. The effects of bipolar GVS on visual verticality were consistent among the groups; however, the effects of bipolar GVS on standing posture were dependent on the stimulus side and hemispheric lesion side. Therefore, we conclude that the effects of bipolar GVS on visual verticality and standing posture differ depending on the polarity and hemispheric lesion side.

In general, the SVV is measured when testing the static otolith function. The SVV often deviates toward the contralesional side in patients with an acute right hemisphere stroke [mean deviation, −5.2° (15); −8.8° (24); −2.2° (10)], which is closely associated with visuospatial neglect, presenting contribution of the higher visuospatial cognition function to the SVV perception. In the present study, contralesional deviation of the SVV during no stimulation in the patients with a right hemisphere lesion was relatively small (mean [SD], −1.0° [2.5°]) (Table 3) compared to that in previous studies (10, 15, 24), and most of the patients showed few symptoms of visuospatial neglect on the line bisection test (Tables 1, 2). The patients included in the present study needed to stand independently without any support. This inclusion criterion induced a sampling bias of patients who had a longer time from stroke onset at the time of the study. None of the patients had an acute stroke, and the time from lesion tended to be longer in the patients with a right hemisphere lesion than in the patients with a left hemisphere lesion. Misperception of SVV often decreases within weeks from the onset of stroke (25).

Contralesional-cathodal vestibular stimulation can reduce the pathological deviation of the SVV in patients with a right hemisphere stroke who have visuospatial neglect (15, 16). In the present study, although there was a trend toward a decrease in SVV deviation with contralesional-cathodal stimulation compared to no stimulation in the patients with a right hemisphere lesion, the differences did not reach statistical significance (Figure 1). This finding may be attributed to the fact described above that the patients in the present study had few symptoms of visuospatial neglect, and thus the deviation of SVV without stimulation was small. Interestingly, the effects of bipolar GVS on SVV when reversing the polarity of the stimulation were similar in all groups: controls, the patients with a left hemisphere lesion, and the patients with a right hemisphere lesion (Figure 1). The application of bipolar GVS was highly likely to result in deviation of the visual vertical away from the cathodal stimulus side (toward the anodal stimulus side), as previously reported in healthy participants (26). The common finding observed in all groups indicated high susceptibility to vestibular stimulation for the perception of the visual vertical regardless of the presence of hemispheric strokes, suggesting a direct link and/or overlap of the neural substrates for processing the visual vertical and mediating a vestibular signal by the stimulation. A functional magnetic resonance imaging (fMRI) study has shown that the bilateral temporo-occipital and parieto-occipital cortical networks associated with cerebellar and brainstem areas were involved in the perception of the visual vertical (27), which was consistent with neuroanatomical studies in patients with stroke (28–30). Recently, some areas in this network have been shown to be involved in vestibular information processing (31).

GVS was used to measure short-latency balance responses in patients with middle cerebral artery stroke standing with their eyes closed (32). Imbalance in activities of the bilateral vestibular afferents evoked by GVS produces a sensation of head movement and compensatory vestibulo-ocular and vestibulospinal reflexes (19). In the present study, vestibular stimulation lasted for 30 s, and the patients were required to stand with their eyes open for the stimulation period. Since sensory reweighting involves control of a stable stance in various environments (33), the procedures in the present study allowed us to investigate the ability to adjust balance control against imbalance in the bilateral vestibular afferents using other sensory modalities, including visual inputs. In contrast to the findings on modulation of visual verticality, where the effect of reversing the polarity of bipolar GVS on SVV was similar in all the groups, asymmetry in the standing posture was significantly reduced in only the patients with a right hemisphere lesion (Figure 2), even though the weight-bearing asymmetry during no stimulation did not differ between patients with lesions in opposite hemispheric sides (Table 3). These findings reveal that it is difficult for patients with right brain damage to maintain their preferred standing posture under modulation of visual verticality evoked by bipolar GVS, unlike patients with left brain damage or healthy controls. As it has been reported that patients with peripheral vestibular dysfunction showed dramatically inability to appropriately suppress the influence of visual and proprioceptive inputs (34), the vestibular function to appropriately suppress other sensory inputs might deteriorate after right brain damage. The difference in postural responses between the lesion sides suggests that the right hemisphere is likely to have a dominant role in the vestibular inhibitory control of the influence of visual verticality on standing posture, which is consistent with the dominance of the non-dominant hemisphere for vestibular function (35). For clinical measurements, the application of bipolar GVS can clarify whether the vestibular system has neural redundancy after stroke to suppress any effects of the stimulation, including modulation of the visual verticality, on balance. The response of the standing posture to bipolar GVS in the present study was similar to that observed in the previous study using caloric vestibular stimulation, showing that cold caloric vestibular stimulation on the contralesional side reduced postural asymmetry in patients with stroke, predominantly in those with right hemisphere lesions (17), although the mechanism of caloric vestibular stimulation differs from that of GVS. For the clinical use of vestibular stimulation, GVS intensity and duration would be easier to control than caloric vestibular stimulation, reducing adverse effects.

Regarding the stimulus side, a previous study (32) did not observe any polarity-dependent effects of GVS on sway response when patients with middle cerebral artery stroke stood with their two limbs equally loaded and their eyes closed. Contrary to their study (32), we observed polarity-dependent effects on standing posture in patients with a right hemisphere stroke. The ipsilesional-cathodal vestibular stimulation, but not the contralesional-cathodal vestibular stimulation, reduced weight-bearing asymmetry (Figure 2). Since patients always sway away from the cathodal side (32), and the weight-bearing on the ipsilesional limb during no stimulation can decrease to achieve an equilibrium with weight-bearing on the bilateral limbs (Table 3), the relationship between the direction of sway evoked by ipsilesional-cathodal vestibular stimulation and the biased distribution of weight-bearing on the two limbs during no stimulation may be important in understanding the stronger effects observed during the ipsilesional-cathodal vestibular stimulation compared to that observed during the contralesional-cathodal vestibular stimulation. The responses of the standing posture to the stimuli were probably related to the responses of the hemispheres to the stimuli, as vestibular information processing requires multisensory signal integration occurring at structures from the vestibular nuclei to the cortices via thalamic relay (36–46) and middle cerebral artery stroke may result in disruption of the cortico-bulbar projection involving the vestibular control of balance (32). By analyzing fMRI in healthy individuals receiving GVS, more pronounced activation patterns were found in the right hemisphere irrespective of the stimulus side (46, 47). In addition, there were different activation patterns between the hemispheres when GVS with reversed polarity was applied; the right-cathodal vestibular stimulation (the ipsilesional-cathodal vestibular stimulation in the patients with a right hemisphere lesion) induced increased neural activity compared to no stimulation in the cortices involving vestibular processing in only the right hemisphere, whereas the left-cathodal vestibular stimulation (the contralesional-cathodal vestibular stimulation in the patients with a right hemisphere lesion) led to increased neural activity in these areas bilaterally (46, 47). These patterns were considered to arise from two determinants: first, the dominance of the right hemisphere for vestibular processing and second, the stimulated side with the stronger activation ipsilateral to the stimulation (35). In the present study, the two determinants might be responsible for the polarity-dependent effects of bipolar GVS on weight-bearing asymmetry in the patients with a right hemisphere lesion because the right-cathodal vestibular stimulation (the ipsilesional-cathodal vestibular stimulation in the patients with a right hemisphere lesion) can affect vestibular information processing occurring dominantly in the right hemisphere more than in the left-cathodal vestibular stimulation (the contralesional-cathodal vestibular stimulation in the patients with a right hemisphere lesion).

Neuromodulation evoked by vestibular stimulation is essential to understand the responses of visual verticality and standing posture in patients with stroke. Signals evoked by the stimulation are transmitted through the vestibular afferents to the vestibular nuclei. The vestibular nuclei send projections to the cerebellum and are critically involved in the vestibulo-ocular reflex or the vestibulocollic and vestibulospinal reflexes. Furthermore, the vestibular nuclei integrate vestibular, cerebellar, visual, and somatosensory signals (44) and project to the thalamic nuclei associated with other modalities where vestibular information processing is considered to occur as well (48). The parieto-insular vestibular cortex receives its main thalamic input in non-human primates (41) and has neuronal responses to multi-modal stimulation (37, 38), while the human homolog of the parieto-insular vestibular cortex comprises multiple areas (45) and its location is still inconclusive. Results of human imaging studies pointed to a distributed cortical network revealed by GVS, including regions in the parietal, frontal, temporal, and insular cortices (36, 46, 47). In the present study, bipolar GVS signals mediated by vestibular pathways modulated visual verticality in all participants (Figure 1), while the signals modulated standing posture in only the patients with a right hemisphere lesion (Figure 2). Therefore, the right hemispheric stroke (Table 1) is likely to directly or indirectly interrupt bipolar GVS signal integration with other modalities occurring at the multi-level vestibular network to maintain stable stance under modulation of visual verticality. We emphasize that bipolar GVS can clarify the function of the vestibular network in patients with stroke to suppress and integrate unexpected neuromodulation evoked by it, which cannot be uncovered by just identifying the lesion location or observing the neurological symptoms.

There are some limitations of the present study. First, we did not test whether our sample size of 33 participants was sufficient to answer the study questions. Instead, we calculated the absolute effect size between the two stimulation conditions in each group (see Materials and Methods). The effect size on SVV was almost large in the patients with a right hemisphere lesion and was large in the patients with a left hemisphere lesion and in the controls. In contrast, the effect size on standing posture was almost large in only the patients with a right hemisphere lesion; it was small in the patients with a left hemisphere lesion and in the controls. These findings support our conclusions irrespective of the sample size. Second, we did not examine the effects of bipolar GVS on SVV and standing posture in relation to brain lesions, although information regarding the stroke area is provided in Table 1. A further study with detailed imaging analyses of a large number of samples will clarify the neural correlates of visual vertical, postural balance, and vestibular function. Third, we did not assess the semicircular canal function and the otolith function in detail. GVS has an influence on these functions, therefore, we cannot exclude the possibility that the conditions of these functions affected our findings. Finally, we focused on the effects of bipolar GVS on only the visual verticality and weight-bearing. It is worth noting that previous studies have reported that egocentric vertical perception (the longitudinal body axis), but not the allocentric vertical perception (SVV), is related to weight-bearing asymmetry after adjusting for motor and sensory functions (4, 5). Furthermore, compared to the deviation of the visual vertical, the deviation of the postural vertical was more closely related to postural disorders (49). Further studies are required to explore the effects of GVS on egocentric vertical and the relationship between egocentric/allocentric vertical and weight-bearing asymmetry. This study could not address the causal relationship between verticality and standing posture. However, we can state that there is a dissociation of responses of the SVV and weight-bearing to bipolar GVS based on the polarity of the stimulation and hemispheric lesion side. This might be a key to understanding the neural mechanisms underlying balance disorders after stroke.

## Conclusions

We demonstrated that manipulation of the vestibular system using bipolar GVS has an influence on visual vertical perception and standing posture depending on the polarity of the stimulation and hemispheric lesion side. The response of the standing posture under the modulation of visual verticality may represent an intrinsic characteristic of patients with right brain damage who have reduced neural redundancy in the vestibular inhibitory system. This will improve the understanding of the neural mechanisms that underlie balance disorders after stroke and the development of effective therapy and rehabilitation in the future.

## Data Availability Statement

The raw data supporting the conclusions of this article will be made available by the authors, without undue reservation.

## Ethics Statement

The studies involving human participants were reviewed and approved by the Tokyo Bay Rehabilitation Hospital Ethics Committee. The patients/participants provided their written informed consent to participate in this study.

## Author Contributions

TT designed the research, performed experiments, and analyzed data. TT, KK, and YO discussed the results. TT and YO wrote the paper. All authors contributed to the article and approved the submitted version.

## Conflict of Interest

The authors declare that the research was conducted in the absence of any commercial or financial relationships that could be construed as a potential conflict of interest.

## Publisher's Note

All claims expressed in this article are solely those of the authors and do not necessarily represent those of their affiliated organizations, or those of the publisher, the editors and the reviewers. Any product that may be evaluated in this article, or claim that may be made by its manufacturer, is not guaranteed or endorsed by the publisher.
